# A machine learning approach to predict *in vivo* skin growth

**DOI:** 10.21203/rs.3.rs-4246629/v1

**Published:** 2024-04-18

**Authors:** Matt Nagle, Hannah Conroy Broderick, Adrian Buganza Tepole, Michael Fop, Aisling Ní Annaidh

**Affiliations:** 1 SFI Centre for Research Training in Foundations of Data Science, University College Dublin, Belfield, Dublin 4, Ireland.; 2 School of Mechanical and Materials Engineering, University College Dublin, Belfield, Dublin 4, Ireland.; 3 School of Mechanical Engineering, Purdue University, West Lafayette, USA.; 4 School of Mathematics and Statistics, University College Dublin, Belfield, Dublin 4, Ireland.; 5 Charles Institute of Dermatology, University College Dublin, Belfield, Dublin 4, Ireland.

**Keywords:** skin growth, tissue expansion, machine learning, artificial neural network, finite element simulation, elastic wave propagation

## Abstract

Since their invention, tissue expanders, which are designed to trigger additional skin growth, have revolutionised many reconstructive surgeries. Currently, however, the sole quantitative method to assess skin growth requires skin excision. Thus, in the context of patient outcomes, a machine learning method which uses non-invasive measurements to predict *in vivo* skin growth and other skin properties, holds significant value. In this study, the finite element method was used to simulate a typical skin expansion protocol and to perform various simulated wave propagation experiments during the first few days of expansion on 1,000 individual virtual subjects. An artificial neural network trained on this dataset was shown to be capable of predicting the future skin growth at 7 days (avg. ***R***^**2**^ **= 0.9353**) as well as the subject-specific shear modulus (***R***^**2**^ **= 0.9801**), growth rate (***R***^**2**^ **= 0.8649**), and natural pre-stretch (***R***^**2**^ **= 0.9783**) with a very high degree of accuracy. The method presented here has implications for the real-time prediction of patient-specific skin expansion outcomes and could facilitate the development of patient-specific protocols.

## 1 Introduction

Human skin is a remarkable organ, it acts as a durable barrier, safeguarding us from the environment while maintaining the flexibility needed to facilitate effortless daily movement. Furthermore, if applied slowly, the skin demonstrates an incredible ability to facilitate extreme deformations by increasing its surface area to reduce the mechanical load [1]. We see many examples of this in day to day life, for example the abdominal skin accommodating extreme deformations during significant weight gain or the stretching of the earlobe through the use of gauges of increasing size for aesthetic or cultural reasons [2]. If stretched beyond its physiological limit for long enough, this surface area increase will be irreversible and referred to as *skin growth* which can be seen, for example, in the laxity of the skin after weight loss.

This phenomenon of skin growth (i.e. irreversible deformation) triggered by mechanical stretch was first exploited for soft tissue reconstruction by Neumann in 1957, who used a subcutaneous rubber balloon to reconstruct an ear [3]. This tissue expansion procedure was further refined by Radovan for defect/scar management and breast reconstruction post mastectomy [4] [5]. While other alternatives to the method have been explored, for example by Austad and Rose with a self-inflating tissue expander [6], the Radovan model of surgeon-controlled inflation has become the most popular. This is due to advances in more reliable injection ports to inflate the expander and reduced risk of tissue necrosis if the expander ruptures [7]. Tissue expansion has since revolutionised reconstructive skin surgeries and is commonly used to repair birth defects [8], burn injuries [9], perform breast reconstructions post mastectomy [7] [10], and perform scalp, ear and nose reconstruction [7].

Whereas other reconstructive approaches try to replace the skin using a donor site, tissue expansion presents the opportunity to grow extra tissue adjacent to where it is required. The additional tissue provides a good match for skin colour, thickness, texture, and sensation while minimising scarring and rejection [11]. While the tissue usually exhibits epidermal thickening and dermal thinning during expansion [12] [13] [14], samples of human skin taken some time after completion of expansion show that this is reversible and the blood supply to the expanded tissue is normal [13] [15]. The expansion process is cost-effective, repeatable, and avoids cosmetic downsides associated with having a donor site [12].

The first step in the current Radovan method of surgeon-controlled tissue expansion involves selection of the expander shape, size and its location. Subcutaneous placement of the expander is usually performed while the patient is under general anaesthesia [13]. This procedure typically involves dissection of subcutaneous pockets located adjacent to the skin defect. The injection ports to inflate the expander may either be buried nearby or left outside for ease of injection [16]. To ensure wound healing has progressed sufficiently, the inflation process is usually started two to four weeks after expander insertion [10] [13]. The expander is then filled with saline at periodic intervals, usually once per week, stretching the skin and thereby stimulating tissue growth. Due to the current dearth of methods to objectively measure skin growth *in vivo*, clinicians rely on their experience and simple heuristic techniques such as visual inspection of skin colour, capillary refill, skin palpation, and patient comfort to determine the volume and frequency of the expansion [13] [10]. Once sufficient new skin is generated, the expander can be removed and the new skin flap can be used for reconstruction.

Unfortunately, despite its numerous advantages, tissue expansion is limited by the lack of *in vivo* methods to objectively measure skin tension and growth. The success of the procedure relies on the personal experience of the clinician to determine the optimum inflation protocol for the patient [17] [8]. Common complications of the procedure include: haematoma, infection, inadequate excess skin to reconstruct the defect, exposure of the expander, implant failure, skin flap ischaemia, and skin necrosis [13] [18] [17]. Initial reports of complication rates were very high, between 20% and 40% in paediatric patients [19], and while more modern reports indicate slightly lower complication rates, they unfortunately remain unacceptably high. For example, 8.9% of patients experience skin necrosis from breast tissue expansion following a mastectomy [20], 25% of patients require treatment for infection during breast reconstruction, and 18% of patients required reoperation within 3 months [21].

The inability to non-invasively measure *in vivo* tension and skin growth is a contributing factor to the high complication rates for patients. Upon excision, skin is known to contract due to the removal of the natural *in vivo* pre-stretch [22] [23] [24]. Since the magnitude of the natural pre-stretch of a patient’s skin is currently unknown, clinicians must fill the expander until the skin flap is 30% to 50% longer than necessary to account for this contraction [13]. For the same reason, it is common practice to overexpand the expander to 110% to 120% of the manufacturer-specified volume [10]. This can lead to either under-inflation of the expander and not enough excess skin to cover the defect, or over-inflation, causing unnecessary discomfort for the patient and risking exposure of the expander, ischaemia, and skin necrosis due to excess stretch. Similarly, the *in vivo* growth of the skin is unknown, making it difficult to determine the optimum inflation protocol. Clinicians must use heuristic techniques to assess whether the skin can bear further expansion. The goal of this study is to present a non-invasive method to measure important material properties of the skin, including natural pre-stretch, stiffness, and growth rate, and to predict the quantity of skin growth 7 days after expansion. Motivated by our previous work [25] [26], a non-invasive wave propagation technique is used in the following procedure:

1. Development of a three-dimensional finite element (FE) model to simulate skin growth through tissue expansion, followed by non-invasive wave propagation measurements.
2. Creation of a large database of 1,000 simulated test cases representative ofa human population.
3. Development of a machine learning (ML) model capable of solving the ill-posed and inverse problem of determining *in vivo* natural pre-stretch, skin stiffness, growth rate, and skin growth 7 days after expansion from elastic wave measurements.

## 2 Results

### 2.1 FE Results

A three-dimensional FE simulation was developed, simulating the complex deformation and growth field caused by the inflation of a typical 60 cc rectangular tissue expander. First, the skin is subjected to an isotropic pre-stretch to obtain the natural *in vivo* state. Then, the tissue expander is used to deform the skin past the natural pre-stretch. The skin is held in this pre-stretched state, allowing it time to grow. Finally, an applied perturbation generates a wave that propagates along the surface of the skin. The normal displacement of the skin at a surface node 5 mm away from the impact is stored, which constitutes our wave propagation measurement; see Section 4.1 for more details. As our goal is to predict skin growth 1 week after expansion, three time points were determined as being of interest for wave propagation measurements: pre-expansion when the skin is in its baseline *in vivo* state, immediately post-expansion, allowing no time for skin growth, and halfway through the typical 1-week check-in period at day 3.5.

**Fig. 1:**
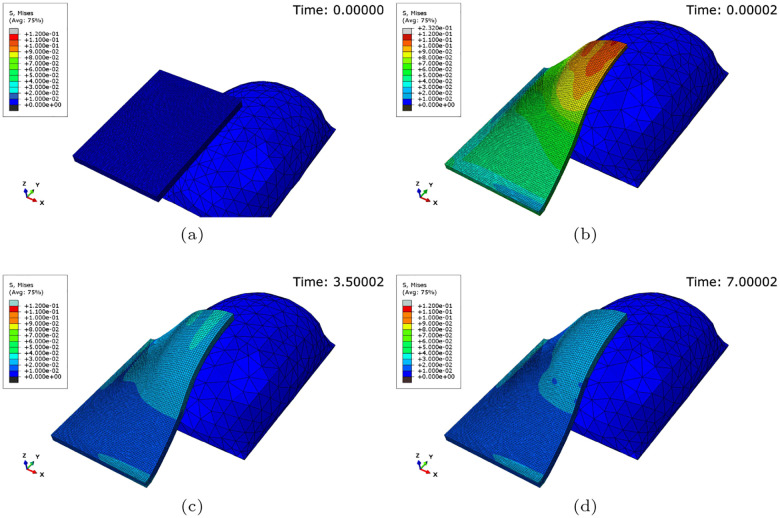
Evolution of the Von Mises stress (MPa) throughout the FE simulation: (a) The stress-free skin block at the start of the simulation, (b) the non-homogeneous stress field immediately after the deformation from the 60 cc pre-inflated expander, (c) the stress field after 3.5 days of growth, and (d) the stress field after 7 days of growth. Neo-Hookean material with a shear modulus *μ* = 0.058335 MPa, a density *ρ* = 1120kgm^−3^, a growth rate *k* = 1.2day^−1^ and a natural pre-stretch *θ*_nat_ = 1.125 (12.5% extension). Note that, taking advantage of the symmetry of the rectangular expander, it was only necessary to model a quarter of the skin.

Over time, as the skin grows, the stress in the skin decreases; see Figure 1. This process can be expressed as the transition between elastic deformation and irreversible deformation, corresponding to skin growth. The irreversible nature of skin growth is demonstrated explicitly in supplementary Figure 2. Note that, taking advantage of the symmetry of the rectangular expander, it was only necessary to model a quarter of the skin.

**Fig. 2:**
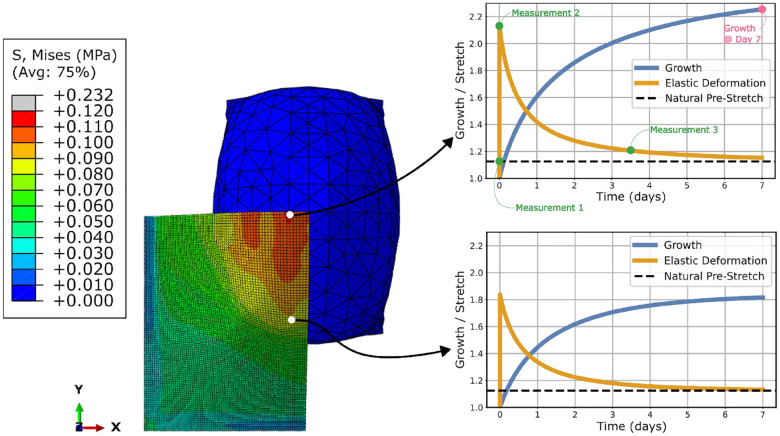
Growth and elastic deformation of the skin as a function of time. Note that the stress and growth fields are both non-homogeneous. As the elastic deformation close to the apex of the expander (upper right of the skin block in the current orientation) is much higher, that region will also experience more growth before the elastic deformation has returned to the natural pre-stretch value. Conversely, the elastic deformation further away from the apex is lower, resulting in reduced growth.

When the skin is stretched past the natural pre-stretch by the expander, skin growth is triggered, causing the elastic deformation to decay back towards the natural pre-stretch value; see Figure 2. Note that the growth is highest in the regions that experience the most elastic deformation and lowest in the regions that experience the least elastic deformation.

To create a large database of test cases representative of a human population, a Latin hypercube sampling technique was used to generate 1,000 unique sets of material parameters (shear modulus *μ*, growth rate *k*, natural pre-stretch *θ*_nat_ and density *ρ*) using reasonable parameter ranges; see Section 4.2. For each of these virtual “subjects”, 3 distinct dynamic wave propagation procedures were completed where the displacement-time curve normal to the surface 5 mm from the impact site was stored for analysis and 1 static simulation was completed to store the growth field at 7 days:

1. The skin was statically stretched to the natural pre-stretch value andmeasurement 1 was performed.
2. The skin was statically stretched beyond the natural pre-stretch value by the expander, held in that stretched configuration for 10^−5^ days (< 1 second), and measurement 2 was performed.
3. The skin remained in that stretched configuration for 3.5 days, and measurement 3 was performed
4. The skin remained in that stretched configuration for 7 days. The resulting growth field of the skin, simplified to be a 5×5 grid of growth values (see Section 4.3), was stored for analysis.

**Fig. 3:**
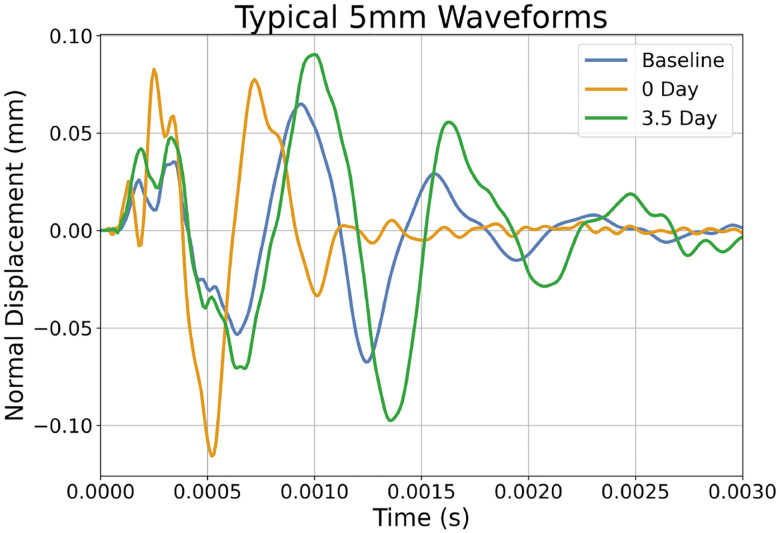
Graph of the displacement normal to the surface of the skin for a node 5 mm away from the impact, generated by the FE simulation. Note, the baseline waveform is shown alongside the 0 days of growth and 3.5 days of growth waveforms. Neo-Hookean material with a shear modulus *μ* = 0.058335 MPa, a density *ρ* = 1120kgm^−3^, a growth rate *k* = 1.2day^−1^ and a natural pre-stretch *θ*_nat_ = 1.125 (12.5% extension).

A typical displacement (normal to the surface of the skin) vs time graph for the wave propagation experiment is shown in Figure 3. A visualisation of how the wave propagation generates the waveforms can be found in supplementary Figure 4. Note that the waveform from day 0 (i.e. just after the expander has been inflated) is shifted to the left of the baseline. This is attributed to the increased tension in the skin, resulting in a faster travelling surface wave. Conversely, note that the waveform after 3.5 days of growth has been shifted to the right, closer to the baseline waveform. This is due to the transition from excess skin tension to skin growth.

This data from the three wave propagation scenarios (from 1,000 individual “subjects”) was then used to predict the simplified 5×5 growth field at the typical 7-day check-in period, along with the subject-specific material properties such as the growth rate of the skin (*k*), the natural pre-stretch (*θ*_nat_), and the stiffness of the material (*μ*).

### 2.2 ANN Results

As outlined in Section 1, the primary goal of this study was to use non-invasive measurements from a wave propagation procedure to predict skin growth and other skin properties of interest. Specifically, an artificial neural network (ANN) was trained to take the baseline, 0 day, and 3.5 day waveforms as inputs, while the target variables were the 25 spatially varying growth values at day 7 from the simplified 5×5 growth field (see Figure 8), the shear modulus *μ*, the growth rate *k*, and the natural pre-stretch *θ*_nat_; see Section 4.3.

**Fig. 4:**
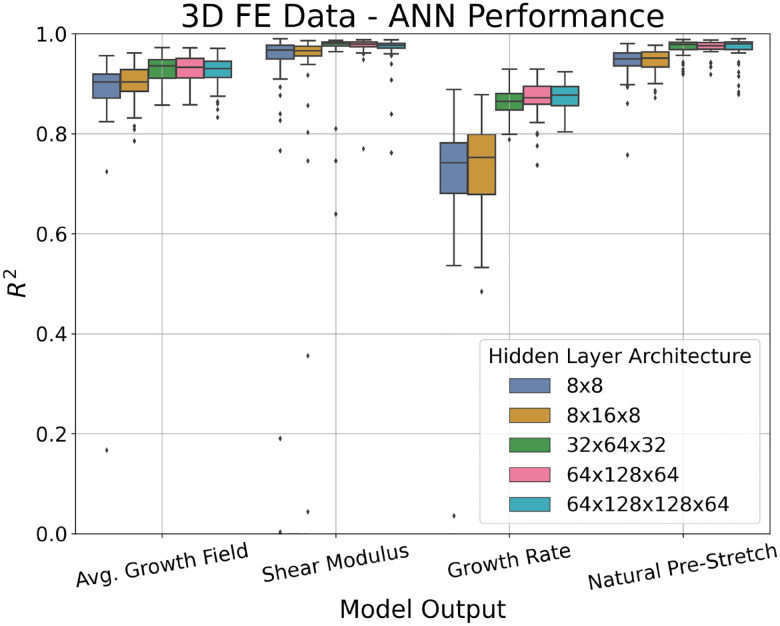
Distribution of the *R*^2^ performance from the 10-fold CV procedure with 5 repeats for different hidden layer architectures for the target variables: 5×5 growth field at 7 days, shear modulus *μ*, growth rate *k*, and natural pre-stretch *θ*_nat_. Note that the average performance for the 25 growth field target values was plotted for visual simplicity. Out of the architectures tested, the 32×64×32 node hidden layer architecture was found to have the best performance.

As the performance of an ANN is dependent on the number of hidden nodes and layers, the architecture of the ANN was tuned using a 10-fold cross-validation (CV) procedure [27]. In this procedure, the dataset is randomly divided into 10 folds of 100 subjects. Subsequently, the ANN is trained on nine folds and tested on the remaining fold. Given the randomness inherent to the data splitting process and the stochastic nature of the ANN weights initialisation, this 10-fold CV procedure was repeated 5 times with different random seeds to ensure robustness and account for random variability in the predictive performance. As a performance metric, the *R*^2^ value [28] computed between the ANN estimated output and the FE simulation output for each of the target variables was calculated on the unseen test set subjects. The distribution of the performance for each of the target variables across various hidden layer architectures is depicted in Figure 4. Note that, for simplicity, the average *R*^2^ value for all 25 growth value predictions was used for visualisation.

We are most interested in maximising the median performance of the growth field and shear modulus predictions. Thus, out of those tested, the hidden layer architecture with the best performance was found to be three fully connected sequential hidden layers with 32, 64 and 32 nodes, respectively. The average performance can be seen in Table 1. These results indicate that the ANN is capable of very accurate predictions of the complex growth field and material properties of the skin using non-invasive measurements from the wave propagation procedure.

**Fig. 5:**
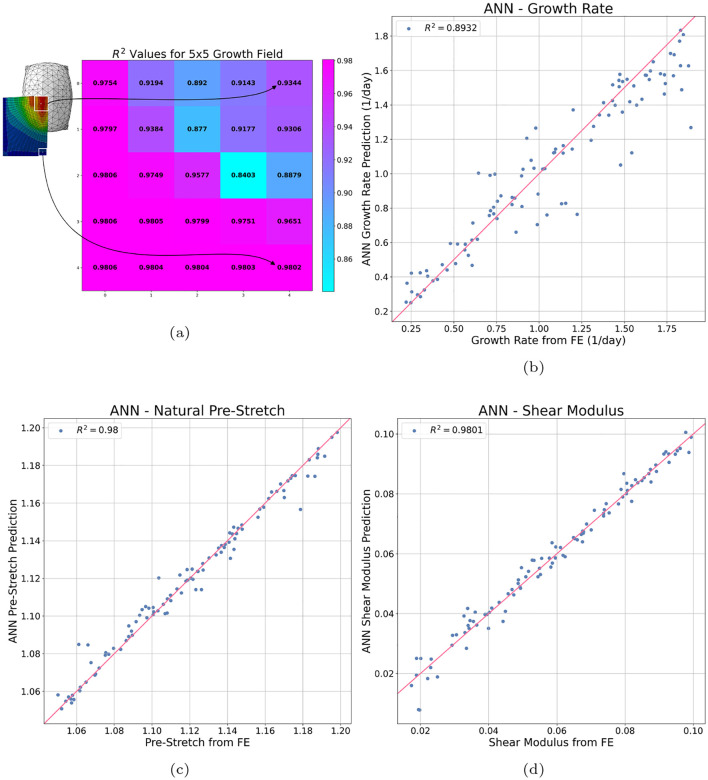
Performance of the ANN model trained on 90% of the dataset and tested on the remaining unseen 10%. For each data point, the x coordinate is the “true” value of the target variable extracted from the FE simulation and the corresponding y coordinate is the ANN prediction of the target variable given the baseline, 0 day, and 3.5 day waveforms for that subject. Note that for simplicity, the 5×5 grid of *R*^2^ values for the growth field predictions has been used in lieu of 25 scatter plots. As shown, the ANN has very high predictive accuracy across (a) the entire growth field, (b) the growth rate, (c) the natural pre-stretch and (d) the shear modulus. Note that (a) represents a quarter model of skin due to symmetry.

To obtain a visual indication of the ANN predictive performance, we examine a specific 90%/10% train-test split, where 900 subjects were used for training the ANN and 100 subjects were held out as an unseen test set. By comparing the predictions of the ANN model to the “true” values from the FE simulation for the unseen test set subjects, we can see that the model is capable of extremely accurate predictions of the skin growth and material properties; see Figure 5. Note that, for ease of visualisation, the *R*^2^ values for the 5×5 growth field predictions are reported instead of 25 separate scatter plots. Overall, we can see the model is capable of very accurate predictions of the growth and material properties. Furthermore, it should be emphasised here that the inputs to these models consist of waveforms at a single measurement location (5 mm from the impact site). Even without prior knowledge of additional locations, the trained ANN demonstrates a remarkable ability to accurately predict the future growth field across the entire region.

**Fig. 6:**
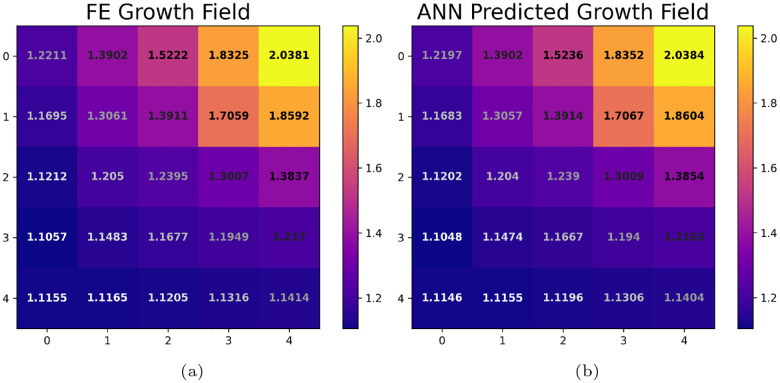
(a) “True” 5×5 growth field at 7 days, (b) Predicted 5×5 growth field at 7 days from the ANN for a randomly selected subject from the unseen test set. Note that the growth predictions agree to within two decimal places for the entire growth field.

Finally, although we can see that the predictive performance of the ANN measured by the *R*^2^ value when predicting the 5×5 growth field at 7 days is satisfactory, (see Figures 5a and 4), it can be difficult to interpret this performance in the context of the actual growth values. To provide some additional visual information, we randomly selected a subject from the unseen test set and compared the “true” 5×5 growth field from the FE simulation to the predicted growth field from the ANN, see Figure 6. Note that the growth predictions at 7 days agree to at least two decimal places for the entire field. This agreement was found to be typical of the subjects in the unseen test set.

A useful metric for surgical applications is the total area of new skin generated by the expansion process. This can be calculated by comparing the new total grown area from the initial area of the skin block. Using this metric, the “true” area of extra skin generated from the 7-day tissue expansion from the FE simulation was calculated to be 700.8960mm^2^, while the predicted extra skin from the ANN model was calculated to be 700.4322mm^2^, an agreement to within 0.066%. We acknowledge that the ANN may pose challenges in quantifying and managing the uncertainty that naturally arises in clinical settings. Nonetheless, the demonstrated predictive performance and the efficiency of the ANN in this study suggest, in theory, the method is accurate and fast enough for real-time clinical use.

## 3 Discussion

A three-dimensional finite element simulation was developed to model skin growth through tissue expansion; see Section 4.1. A simplified two-dimensional finite element was also developed and can be seen in supplementary Figure 1. The same procedure was implemented for the two-dimensional simulations, which led to good predictive performance and allowed for easy interpretation of the irreversible nature of skin growth and the formation of the waveform; see supplementary information. The simulations employed a hyperelastic user-defined material with a previously calibrated growth law [29]; see Section 4.1. The FE model demonstrated behaviour consistent with the existing literature: prolonged mechanical stretch triggers tissue growth [30] [13] [14]. Alternatively, this phenomenon can be described as a transition between the elastic deformation of the skin past the natural pre-stretch to permanent skin growth; see Figures 1 and 2.

Using the FE model, a dataset consisting of 1,000 unique subjects was created. This dataset maps the unique skin material properties and growth field to the waveform obtained from the wave propagation procedure. Using this dataset, an ANN was trained to use the baseline, 0 days of growth, and the 3.5 days of growth waveforms to predict the growth field at 7 days along with the material properties of interest; see Section 4.3.

The ANN was extensively tested and tuned using a cross-validation procedure. Despite the complexity of the data (due to wave reflections, non-homogeneous stress fields, and complex deformation around the expander), the ANN still yielded high predictive performance; see Table 1.

As discussed in Section 1, currently, there exists no non-invasive method to determine skin growth *in vivo*. The sole method to differentiate between elastic stretch and irreversible growth is the excision of tissue [31]. Thus, in the context of patient outcomes, a pre-trained ML model of the form presented here holds significant value. The model demonstrates that non-invasive data obtained from a simple and inexpensive wave propagation device can be used to obtain accurate real-time predictions of future *in vivo* skin growth and other material properties of interest at negligible computational expense.

There are, however, a number of limitations to this approach. As with any ML approach, there is an assumption that the training data is representative of the “true” data-generating process. Therefore, the models presented here may find it difficult to generalise to experimental data, as the training data solely consists of waveforms obtained from FE simulations, which are inherently smooth and noiseless. However, in theory, once some experimental data becomes available, the simulated training dataset could be augmented or altered to mimic the observed noise level and precision of the wave propagation device. Additionally, statistical methods like bootstrapping [32] [27] could be employed to estimate the variability in predictive performance. Moreover, alternative ML methods that can encompass and account for uncertainty [33] could facilitate better generalisation to clinical and experimental data.

Similarly, depending on the limitations of a physical wave propagation device, it may be necessary to perform some additional post-processing of the simulated training data. For example, if the device cannot capture waveforms of the form shown in Figure 3, it may be necessary to use signal processing techniques to extract wave features common to the FE and experimental data. Subsequently, the ML model would need to be retrained.

It should also be noted here that the bounds for the input space (see Table 2) must be selected carefully, as the model will only be able to reliably make predictions in that input space. As such, great care was taken to select ranges consistent with what we expect in observations from *in vivo* human skin; see Section 4.2. However, there is a lack of consensus in the literature for the ranges of some of these material properties (for example, the shear modulus [34]).

While a variety of different strain energy functions have been developed in the literature [35], for simplicity of implementation, in this study a transversely isotropic hyperelastic neo-Hookean framework was used to model *in vivo* human skin; see Section 4.1. As such, the pre-trained ANN may not generalise well to data from other constitutive models. Future work could involve the extension to other material models and exploring full anisotropy.

As discussed in our previous work [26], this general framework could be viewed as an alternative to inverse FE techniques [36] [37] [38]. The benefit of this framework is that almost all computational cost and expertise required is up front. Once the ML model has been trained and validated, it can be deployed with minimal computational cost and expertise, allowing for real-time predictions.

Currently, the choice of expander size and geometry is often based on the preference of the clinician [13], with limited research exploring different expander designs. The framework presented here could be used to explore the effect that expander geometry and surface texture has on the growth field, aiding clinicians in the selection process.

The model presented here is trained to predict the growth field at 7 days resulting from a single inflation from a specific expander geometry and volume (60 cc). Clinically, however, most inflation procedures consist of multiple inflation steps based on clinician experience and heuristic observations. The method proposed here could be used to standardise the first week of treatment for all patients, enabling clinicians to acquire relevant tissue parameters like the growth rate. Using this information, a data-driven subject-specific inflation procedure could be designed, either using patient-specific forward FE simulations or estimating general treatment guidelines, such as a safe volume per inflation based on the stiffness and growth rate of the patient.

In conclusion, existing methods to determine skin growth *in vivo* are heuristic and rely on significant experience of the clinician. This is a contributing factor in poor patient outcomes from reconstructive surgeries. As such, the goal of this study was to propose an *in vivo* method for non-invasively determining pertinent mechanical properties of patient skin and predicting future skin growth using elastic wave measurements. A three-dimensional FE model was developed to simulate skin growth through tissue expansion using a typical 60 cc expander geometry. A large dataset consisting of simulated real-world wave propagation experiments at three stages of the expansion process (baseline, day 0 and day 3.5) was constructed using the FE model. An ANN was implemented to take the non-invasive wave propagation data as inputs and predict the unique material properties, as well as the future growth 7 days after inflation of the expander. An ANN trained on the FE data was shown to have high predictive performance for the growth field (*R*^2^ = 0.9353), shear modulus (*R*^2^ = 0.9801), growth rate (*R*^2^ = 0.8649), and natural pre-stretch (*R*^2^ = 0.9783). To the best of our knowledge, the framework introduced here is the first of its kind, utilising non-invasive *in vivo* measurements that are cheap and easily obtained, in conjunction with modern ML techniques to measure tissue properties and predict future skin growth in real time. Approaches of the form presented here could be of significant use to clinicians to help design patient-specific inflation protocols and design general treatment guidelines, thereby improving patient outcomes.

## 4 Materials and Methods

### 4.1 Finite Element Modelling

**Fig. 7:**
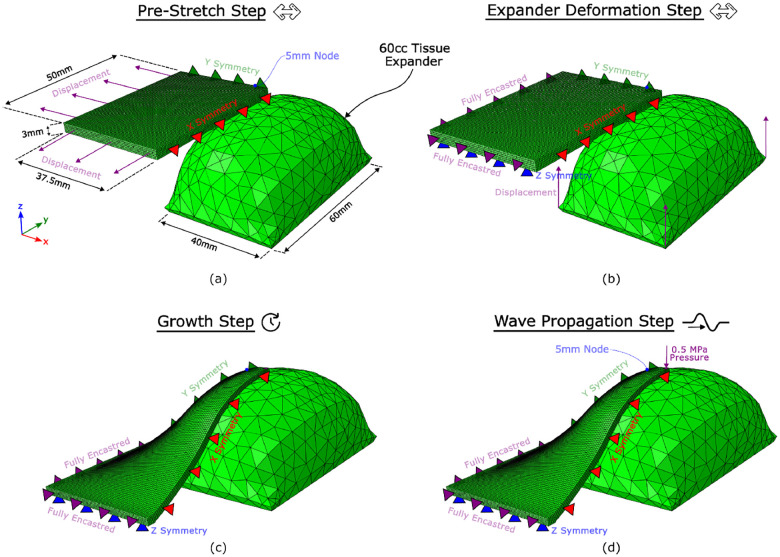
Dimensions and boundary conditions for the FE model of wave propagation. (a) The uniaxial pre-stretch is generated using displacement boundary conditions. (b) The stretch beyond the natural pre-stretch is generated using a typical 60 cc rectangular expander geometry. (c) The skin is held in this stretched configuration to promote growth. (d) The wave is generated by a 0.5 MPa pressure applied for 2 × 10^−5^ s. The normal displacement of a node 5 mm away from the impact site was stored for analysis.

To implement the FE model, the nonlinear FE package Abaqus/Standard (Dassault Systems, Waltham, MA) was used to statically pre-stretch the skin, deform the skin further with the expander using displacement boundary conditions, and hold the skin in the final stretched state to allow time for skin growth. Subsequently, Abaqus/Explicit (Dassault Systems, Waltham, MA) was used to perform the wave propagation procedure where a 0.5 MPa pressure was applied for 2 × 10^−5^ s and a wave was allowed to propagate through the skin. The displacement in all three dimensions of a surface node 5 mm from the impact site was stored, and a displacement normal to the skin’s surface was constructed for analysis. As depicted in Figure 7, the dimensions of the unstretched block are 50mm×37.5mm×3mm. The skin block was discretised into 45,000 C3D8 elements with 53,732 nodes.

In order to simulate the skin growth through tissue expansion process, the skin block was represented by a user-defined hyperelastic material developed in previous works [29] [39]. A neo-Hookean framework was used with strain energy density function:

(1) $$\Psi_{\text{NH}}=\frac{\mu }{2}\left(I_{1}^{\text{e}}-3\right)+\frac{\lambda }{2}{\text{ln}}^{2}\left(J^{\text{e}}\right),$$

where *μ* and *λ* are the Lamé parameters, $I_{1}^{\text{e}}$ is the first strain invariant of the left Cauchy-Green tensor, and *J*^e^ is the volume change [29] [40]. $I_{1}^{\text{e}}$ and *J*^e^ arise from the split of the deformation gradient ***F*** into the growth and elastic contributions, ***F***^e^ and ***F***^g^ respectively [29]:

(2) $$F=F^{\text{e}}F^{\text{g}},$$

with determinants *J*^e^ = det(***F***^e^) and *J*^g^ = det(***F***^g^). Furthermore, assuming only changes in area and no change in thickness:

(3) $$J^{\text{g}}=\theta_{\text{growth }}=\left\|\text{cof}\left(F^{\text{g}}\right)\cdot N\right\|,$$

where *θ*_growth_ is a scalar value representing the total area growth, cof() is the cofactor operator, · is the dot product, and ***N*** is the initial unit normal to the skin. Similarly, elastic changes in area *θ*_elastic_ can be written as [29]:

(4) $$\theta_{\text{elastic }}=\left\|\text{cof}\left(F^{\text{e}}\right)\cdot N\right\|,$$

where ***N*** is the same unit normal as growth increases the size of the differential volume elements but introduces no rotations.

For simplicity of implementation and interpretation, the skin growth in this study was modelled to be transversely isotropic. This is motivated by clinical observations of tissue expansion in paediatric patients [13] and supported by our previous work, which indicated that younger patients have more isotropic skin tension [25]. Han et al. relaxed this assumption and presented an anisotropic version of this model containing two growth rates for the transverse and longitudinal directions [29].

Furthermore, in the literature, using histological analysis, it has been concluded that tissue expansion initiates an increase in surface area (i.e. skin growth) without a corresponding increase in skin thickness [17] [14] [41]. Therefore, in the simulations, the skin was allowed to grow isotropically in-plane, and there was no growth out of plane (i.e. normal to the skin surface).

Skin growth is a complex biological process that can be examined on a number of levels. At the cellular level, skin tension initiates multiple signalling pathways that up-regulate fibroblast mitosis and increases protein synthesis, causing an increase in skin surface area to restore the homeostatic equilibrium state [17]. At the tissue level, mechanical stretch requires force transfer between the different layers of the skin (e.g. the epidermis and the dermis) [42], which initiates cell-cell crosstalk and cell-matrix interactions [17]. However, despite this biological complexity, the overall understanding of mechanical stretch eliciting skin growth allows for the construction of a simplified phenomenological mathematical model for growth: the rate of skin growth is linearly proportional to the mechanical deformation. As our goal is to model *in vivo* skin growth through tissue expansion, we consider any stretch past the natural pre-stretch to elicit skin growth [29]:

(5) $${\dot{\theta }}_{\text{growth }}=k\left(\theta_{\text{elastic }}-\theta_{\text{nat}}\right),$$

where ${\dot{\theta }}_{\text{growth }}$ is the rate of skin growth, *k* is the subject-specific growth rate, *θ*_elastic_ is the elastic deformation, and *θ*_nat_ is the subject-specific natural pre-stretch of the skin.

Our goal is to generate a dataset to train a machine learning model by using a feasible and realistic clinical procedure to generate the data. So, for each block of skin with unique skin parameters, three wave propagation scenarios were performed:

1. The skin was stretched to the natural pre-stretch value and the wave propagation procedure was performed. This represents the situation where a baseline wave propagation measurement is taken when the patient visits the clinic, before the expander is placed (measurement 1).
2. The skin was stretched past the natural pre-stretch value, held in that stretched configuration for 10^−5^ days (< 1 second) and the wave propagation procedure was performed. This scenario represents the immediate wave propagation measurement taken after the expander is placed under the skin and inflated, allowing essentially no time for skin growth to occur (measurement 2).
3. The skin was stretched past the natural pre-stretch value, held in that stretched configuration for 3.5 days and the wave propagation procedure was performed. This scenario represents the measurement taken when the patient returns to the clinic halfway through the typical 1-week check-in period, after 3.5 days of skin growth (measurement 3).

In addition, a fourth simulation was performed where the skin was stretched past the natural pre-stretch value, held in that stretched configuration for 7 days, and the growth field was stored. As we are using a supervised ML approach, the data from the three wave propagation scenarios comprise the input variables and the 7-day growth values are the output variables.

### 4.2 Input Space Sampling

As discussed in our previous work [26], for this data-driven approach, sampling from the input space is a critical step as it determines the range over which the model is capable of making accurate predictions. If the ultimate goal is to make predictions from a sample of *in vivo* human subjects, then the ML model needs to be trained on a wide variety of subjects who have unique combinations of skin material properties in the expected ranges for *in vivo* human skin. However, due to the computational complexity of running the FE models, it is also desirable to minimise the number of samples.

As discussed in Section 4.1, a user-defined material capable of simulating skin growth through tissue expansion was employed. It was necessary to explore a four-dimensional input space of *μ*, *k*, *θ*_nat_, and *ρ* (shear modulus, growth rate, natural pre-stretch, and density, respectively). Note that a value of 40 times the shear modulus was used for Lamé’s first parameter, *λ*. For the purposes of this study, we consider the skin blocks to be nearly incompressible.

In the literature, the density of skin *ρ* is often taken to be constant, for example the value 1116kgm^−3^ [43]. However, in this study, to allow for some variation due to hydration, among other factors, this value was allowed to vary by ±5%. In our custom material model, the growth rate is assumed to be linearly proportional to the elastic growth, see Equation 5. As such, the growth rate *k* is a parameter that controls the non-linear growth rate. In the publication from Han et al., the growth rate *k* in porcine skin was predicted to be *k* ∈ [0.02,0.08]hr^−1^ = [0.48,1.92]day^−1^ [29]. During our testing, it was found that growth rates in this range were fast enough for almost all subjects to return to their natural levels of pre-stretch by the end of the 7-day test period. Therefore, to allow for some more variation in the dataset and account for subjects with slower skin growth, for our study the growth rate was expanded to *k* ∈ [0.2,1.92]day^−1^.

The isotropic natural pre-stretch of the skin *θ*_nat_ reported in the literature varies depending on the measurement procedure and tissue type used. Ní Annaidh et al. reported the mean failure strain of excised human skin to be 54% ± 17% [44], Deroy et al. reported contractions in the 10% to 30% range for canine skin [24], Jor et al. reported a maximum skin retraction of approximately 40% for porcine skin [23], and Han et al. reported the average pre-strain field to be mostly in the range of 1 to 1.25 for the control patches in porcine skin [29]. In this study, it was decided to use a reasonably conservative natural pre-stretch range of 5% to 20%. This is because the skin was going to be stretched significantly past this natural pre-stretch by the expander; see Section 4.1. The conservative range for *θ*_nat_ avoids a subject with a large natural pre-stretch being stretched too close to the mean failure strain of excised human skin, where numerical instability may occur in the FE simulations.

Finally, the *in vivo* stiffness of human skin measured by the shear modulus *μ* (or equivalently the Young modulus *E*) has been shown to vary significantly depending on the measurement method. Assuming an incompressible material where the relationship between the shear and Young’s modulus can be expressed as *μ* = *E/*3 [45]: Li et al. reported values of the forearm dermis in the range *μ* ∈ [0.0508,0.0953] MPa using optical coherence tomography [43], Liang and Boppart reported *μ* ∈ [0.0167,0.05] MPa for forearm skin using optical coherence elastograpy [46], and Diridollou et al. reported *μ* ∈ [0.0267,0.0867] MPa. As in our previous work, we selected a reasonably broad range of values between 0.01667 and 0.1 MPa [26]. However, it should be noted here that there is considerable variation in the literature and, as such, no range will encompass all reported values. For example, Park et al. report *μ* ∈ [0.002,0.008] MPa using an indentation-based device which is far below our chosen range. Conversely, Agache et al. report *μ* ∈ [0.14,0.28] MPa using a torsion test, which is above the chosen range.

In order to guarantee good coverage of the input space with a comparatively small number of samples, a Latin hypercube sampling method [47] was used. Specifically, the function “LatinHypercube” from the Python sub-package “scipy.stats.qmc” [48] was used to generate 1,000 unique sets of material parameters (*μ*, *k*, *θ*_nat_, and *ρ*) using the parameter ranges in Table 2.

### 4.3 Machine Learning - Artificial Neural Network

As discussed in Section 1, accurate, non-invasive measurements of the *in vivo* growth would provide significant benefit to both patients and clinicians. In this study, we propose a surface wave propagation technique as a suitable non-invasive measurement. In our previous work, we have demonstrated that the surface wave speed contains valuable information about the material properties of the skin, which can be extracted [25] [26]. We propose the use of a ML model which can solve the complex, ill-posed problem of inferring the growth and other material properties of the skin from the waveforms. While in principle many different statistical and ML models could be used to perform this task (for example regression models, Gaussian process, random forests, support vector machines, neural networks, etc.), the choice of model depends on the structure of the training data and the specific requirements of the problem at hand.

In our previous work, a Gaussian process (GP) regression model was trained to predict the stress in the principal direction of stretch and the natural pre-stretch of the skin using the speed of two distinct elastic waves as inputs: the supersonic shear wave and the Rayleigh wave [26]. The GP regression model was a suitable choice as it provided both high predictive performance and allowed for uncertainty quantification.

In this study, as our prediction task is inherently more complex, the full shape of the waveforms was used for prediction, rather than just the supersonic and Rayleigh wave speed values. As discussed in Section 4.1, the displacement normal to the skin’s surface for a node at a known distance from the impact site was stored for each FE simulation. Each of these waveforms were then interpolated onto a common grid of 1,001 values to use as input variables. Specifically, the function “InterpolatedUnivariateSpline” from the python sub-package “scipy.interpolate” [48] was used to interpolate the waveforms from the FE simulation onto a regular grid of 1,001 values between 0 and 0.0125 seconds. Thus, our ML models were required to take data points with 3,003 dimensions in input, each corresponding to a displacement of either the baseline, 0 day, or 3.5 day waveforms for a particular time point.

Given the high-dimensional nature of the inputs, GP models were deemed unsuitable, as they have been shown to struggle even in moderate-size settings [49]. Instead, an artificial neural network (ANN) was selected due to its flexibility, its ability to handle high-dimensional inputs, and its high predictive performance. An ANN is a ML model inspired by the structure and functioning of neurons in the human brain. They consist of a number of interconnected layers of artificial neurons, where each neuron receives an input signal and transmits an output to subsequent layers. ANNs are powerful versatile ML models capable of many tasks, including pattern recognition, classification, and regression [50] [51] [52]. We note that uncertainty quantification poses challenges when using standard ANNs [53], as opposed to GP models, which offer a readily available framework for uncertainty. By choosing ANNs, we prioritise predictive performance and computational efficiency over the ability to quantify uncertainty, as their flexibility makes them particularly well-suited for handling the complexities of our prediction task.

**Fig. 8:**
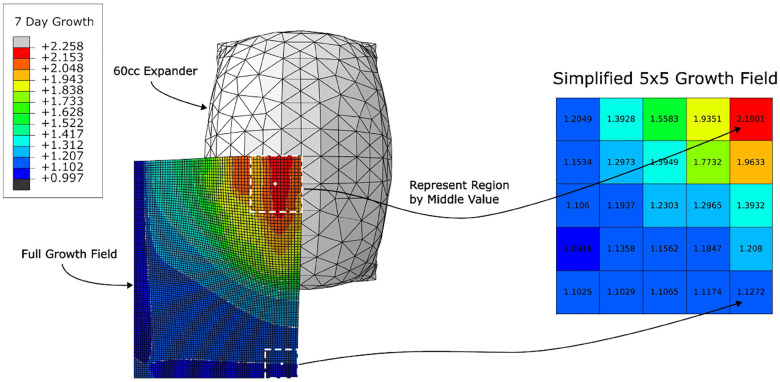
Simplification of the full growth field to a 5×5 growth field (based on the reference configuration dimensions) using the assumption that growth values in a region can reasonably be represented by the growth value of the middle element. Note that it was only necessary to model a quarter of the skin block due to the symmetry of the expander.

The target variables of interest were the material parameters, namely, the shear modulus *μ*, the growth rate *k*, and the natural pre-stretch *θ*_nat_, and the growth field at 7 days. To simplify the prediction task and facilitate visualisation of the growth field, we make the simplifying assumption that, locally, growth values are similar. Hence, the granular growth field at 7 days was simplified to a 5×5 field of growth values (based on the reference configuration dimensions) where the growth value of the element in the centre was taken to be representative of the region, see Figure 8.

**Fig. 9:**
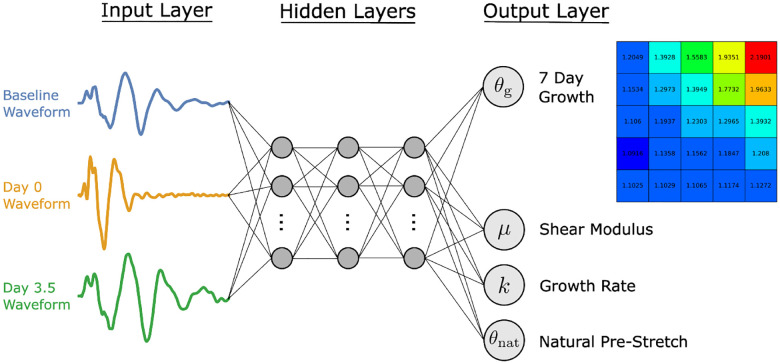
Structure of the ANN. Note that the input layer consists of the baseline, day 0, and day 3.5 waveforms, each of which have been interpolated onto a common grid using 1001 values. Three hidden layers have been shown for the sole purpose of visualisation. The output layer consists of 28 nodes, 25 nodes corresponding to the 5×5 growth grid plus the 3 material parameters.

The general structure of our ANN can be seen in Figure 9. The inputs to the ANN are the baseline, day 0 and day 3.5 waveforms, each of which have been interpolated onto a common grid and represented by 1,001 equidistant points. Our target variables are the shear modulus *μ*, the growth rate *k*, the natural pre-stretch *θ*_nat_, and the 5×5 growth field at 7 days.

The ANN was implemented using the high-level application programming interface (API) Keras using the TensorFlow platform [54] [55]. The architecture of the ANN was tuned as part of the model selection process, see Section 2.2.

## Figures and Tables

**Table 1:** Median *R*^2^ and standard deviation of *R*^2^ from the 10-fold CV procedure with 5 repeats for the ANN trained on the FE data with hidden layer architecture consisting of 32, 64 and 32 nodes respectively.

Target Variable	Median *R*^2^ from 10-fold CV	Standard Deviation of *R*^2^ from 10-fold CV
Avg. Growth Field at 7 days (*θ*_*g*_)	0.9353	0.0270
Shear Modulus (*μ*)	0.9801	0.0621
Growth Rate (*k*)	0.8649	0.0301
Natural Pre-Stretch (*θ*_nat_)	0.9783	0.0196

**Table 2:** Material property ranges used for the input space.

Material Property	Range	Units
*μ*	[0.01667, 0.1]	MPa
*k*	[0.2, 1.92]	day^−1^
*θ*_nat_	[1.05, 1.2]	
*ρ*	[1060.2, 1171.8]	kg m^−3^

## Data Availability

The datasets generated and analysed during the current study are available from the corresponding authors (M.N., M.F. and A.N.A) on reasonable request.
